# Comparison of the Whole-Plastome Sequence between the Bonin Islands Endemic *Rubus boninensis* and Its Close Relative, *Rubus trifidus* (Rosaceae), in the Southern Korean Peninsula

**DOI:** 10.3390/genes10100774

**Published:** 2019-10-02

**Authors:** JiYoung Yang, Koji Takayama, Jae-Hong Pak, Seung-Chul Kim

**Affiliations:** 1 Department of Biology, Research Institute for Dok-do and Ulleung-do Island, School of Life Sciences, Kyungpook National University, 80 Daehak-ro, Buk-gu, Daegu, Gyeongsangbuk-do 41566, Korea; whity@daum.net; 2 Department of Botany, Graduate School of Science, Kyoto University, Oiwake-cho, Kitashirakawa, Sakyo-ku, Kyoto 606-8502, Japan; takayama@sys.bot.kyoto-u.ac.jp; 3 Department of Biological Sciences, Sungkyunkwan University, 2066 Seobu-ro, Suwon, Gyeonggi-do 16419, Korea

**Keywords:** *Rubus boninensis*, *Rubus trifidus*, Rosaceae, Bonin Islands, anagenetic speciation, plastome

## Abstract

*Rubus boninensis* is a rare endemic species found on the Bonin Islands with a very restricted distribution. It is morphologically most closely related to *Rubus trifidus*, occurring widely in the southern Korean peninsula and Japan. This species pair provides a good example of anagenetic speciation on an oceanic island in the northwestern Pacific Ocean—*R. trifidus* as a continental progenitor and *R. boninensis* as an insular derivative species. In this study, we firstly characterized the complete plastome of *R. boninensis* and *R. trifidus* and compared this species pair to another anagenetically derived species pair (*R. takesimensis–R. crataegifolius*). The complete plastome of *R. trifidus* was 155,823 base pairs (bp) long, slightly longer (16 bp) than that of *R. boninensis* (155,807 bp). No structural or content rearrangements were found between the species pair. Eleven hotspot regions, including *trnH/psbA*, were identified between *R. trifidus* and *R. boninensis*. Phylogenetic analysis of 19 representative plastomes within the family Rosaceae suggested sister relationships between *R. trifidus* and *R. boninensis*, and between *R. crataegifolius* and *R. takesimensis*. The plastome resources generated by the present study will help elucidate plastome evolution and resolve phylogenetic relationships within highly complex and reticulated lineages of the genus *Rubus*.

## 1. Introduction

The Bonin Islands, also known as the Ogasawara Islands, consist of 25 small islands (>0.1 km^2^) and many islets scattered in the region of 24°14’–27°44’ N and 140°52’–142°15’ E, and are located approximately 1000 km directly south of the Japanese archipelago [1,2]. The Bonin Islands consist of two island groups, i.e., Ogasawara Group (Hahajima, Chichijima, and Mukojima) and Volcano Group (Kitaiwojima, Iwojima, and Minamiiwojima). The Ogasawara Group is a group of three aggregated islands, and are aligned from south to north, i.e., Hahajima, Chichijima, and Mukojima. Although they were formed during the Paleogene period, their uplift started in the Pleistocene epoch, exposing the landmass above the sea level before the middle Pleistocene epoch [3,4]. Therefore, organisms presumably started colonization from the late Pliocene to early Pleistocene epochs [5,6,7]. Of the 369 indigenous vascular plant species, approximately 40% of them are endemic to the Bonin Islands, originating from the surrounding continental regions, including southeastern Asia, Taiwan, and the Japanese mainland, primarily via allopatric speciation [8,9,10,11]. A recent study of *Liparis hostifolia* (Orchidaceae) also suggested that some endemic taxa likely originated from temperate East Asia, including Japan, Korean Peninsula, China, and Russia [12]. Furthermore, some endemic groups (e.g., land snail; genus *Mandarina*) have undergone accelerated morphological and ecological divergence during adaptive radiations within the archipelago [6,7]. The high plant endemism of the Bonin Islands makes this archipelago an ideal setting for investigating allopatric speciation and adaptive radiation. 

Unlike the Ogasawara Group of the Bonin Islands, some islands in the northwestern Pacific Ocean, such as the Izu Islands, the Volcano Group of the Bonin Islands, and the Northern Marianas Islands, are considered geologically to be much younger. In particular, the Volcano Group of the Bonin Islands is a group of three islands (i.e., Kitaiwojima, Iwojima, and Minamiiwojima, aligned from north to south) situated south of the Ogasawara Group of the Bonin Islands and are geologically much younger, from an approximate age of 140,000 years for Kitaiwojima to 30,000 years for Minamiiwojima [13]. Based on the floristic surveys of Minamiiwojima, 135 vascular plant species have been recorded, nine of which are considered to be endemic to the Volcano Group of the Bonin Islands [12,14]. Approximately 70% of vascular plants are commonly distributed on two other islands of the Volcano group, and approximately 70% also occur on the Ogasawara Group of the Bonin Islands [15]. In addition, closely related congeneric vascular plant species often do not occur on the same Volcano Group Islands, suggesting rare additional speciation events within the islands and a primary role of geographical isolation for the origin of endemic species on the Volcano Islands [9,10,11]. Compared to other oceanic archipelagoes [16], the endemic species of the Bonin Islands (a total of 118 endemic species) presumably evolved via anagenetic speciation (53%) and the remaining (47%) by cladogenetic speciation.

Of the several woody representative genera of the family Rosaceae occurring on the Bonin Islands (i.e., *Osteomeles*, *Photinia*, *Rhaphiolepis*, and *Rubus*), the genus *Rubus* is of great interest, given its diversity and rarity on the islands. Three species of *Rubus* occur on the Bonin Islands: *R. boninensis*, *R. nishimuranus*, and *R. nakaii*. All but one species, *R. nishimuranus* (subgenus *Idaeobatus*), belong to the subgenus *Anoplobatus*. *Rubus nakaii* is one of the critically endangered (CR) species, with rare but wide distribution on the Bonin Islands, while *R. nishimuranus* is a common indigenous species. *Rubus nakaii* differs primarily from *R. trifidus* and *R. boninensis* by having a solitary nodding inflorescence or two flowers instead of an erect corymbose inflorescence [17,18]. *Rubus trifidus* occurs widely in the southern Korean peninsula and along the Pacific coastal area of the Japanese archipelago (Shikoku, Kyushu, and the northern range limit of Honshu-Aomori Prefecture). In particular, *R. boninensis*, the focus of our interest, is a rare endemic plant restricted to the Volcano Islands (Kitaiwojima and Minamiiwojima) and shows limited distribution in the Bonin Islands [18]. Owing to its narrow geographic distribution and poor documentation, very little information on the biology and evolutionary relationship of *R. boninensis* is known. Although the phylogenetic position of *R. boninensis* has never been formally determined, morphologically it is most closely related to *R. trifidus*. However, the former can be distinguished from the later based on a few diagnostic features, such as tri-lobed immature leaves, 3–5 cleft or simple leaves, glandular calyx tube and pedicels, and red drupelets at maturity [18,19]. Our global-scale phylogenetic framework of the genus *Rubus* [20] clearly demonstrated that *R. trifidus* is sister to *R. boninensis*, suggesting a relationship of a continental progenitor and anagenetically derived insular endemic species, respectively. However, as a precise continental source area (i.e., either Japanese archipelago or Korean peninsula, given its native distribution in these areas), the relationships among populations of *R. trifidus* and patterns of genetic diversity and differentiation of *R. boninensis* compared to that of *R. trifidus* (a putative continental progenitor) are yet to be determined.

Resembling the species pair *R. boninensis*–*R. trifidus* as an example of anagenetic speciation found in the northwestern Pacific Ocean, a different pair of progenitor and derivative species via anagenetic speciation can be found on Ulleung Island in East Sea/Sea of Japan, located between the Korean peninsula and the Japanese archipelago. *Rubus takesimensis* (subgenus *Idaeobatus*) is the only endemic species of *Rubus* to Ulleung Island, which is an oceanic volcanic island with an estimated age of 1.8 Myr. Compared to its continental progenitor, *R. crataegifolius*, which occurs rather widely in northeastern Asia (China, Japan, Korea, and Russian Far East), *R. takesimensis* is characterized by a lack of prominent prickles (i.e., loss of defense mechanism) and an overall large status of plants (i.e., insular gigantism) as a response to release from selection pressure of herbivores and due to the fact of its moderate insular climatic setting, respectively. Ulleung Island is known for unusually high levels of anagenetic speciation (at least 88% of vascular endemic species), mainly driven by a lack of vegetation heterogeneity, younger island age, and low elevation [16]. Recently, we demonstrated a sister relationship between this continental progenitor (*R. crataegifolius*) and insular derivative (*R. takesimensis*) species pair and investigated the population genetic structure among them [20,21]. In addition, we compared the complete plastome sequences of *R. crataegifolius* and *R. takesimensis* and characterized their molecular evolution, identifying mutational hotspot regions [22]. The example of anagenetic speciation of *Rubus* found in East Sea/Sea of Japan, *R. crataegifolius*–*R. takesimensis*, and another example found in the northwestern Pacific Ocean, *R. trifidus*–*R. boninensis*, could be an ideal system to investigate genome evolution of organelles during anagenetic speciation on ocean islands.

In this study, we determined two complete plastome sequences of the insular derivative, *R. boninensis*, and the continental progenitor, *R. trifidus*, in the northwestern Pacific Ocean, and compared them to two previously reported plastomes of an anagenetically derived species pair in the East Sea/Sea of Japan. This allowed us to characterize the plastome sequences of two anagenetically derived species in different oceanic islands and to reveal any molecular changes occurring during anagenetic speciation. In addition, we hoped to identify mutation hotspots in the plastomes of *R. boninensis* and *R. trifidus* belonging to subgenus *Anoplabatus*. Such plastome hotspot regions could then be utilized as efficient maternally inherited molecular markers for phylogeographic and population genetic study of the *Rubus* species belonging to subgenus *Anoplabatus*. Lastly, this study aimed to develop simple sequence repeat (SSR) markers based on *R. boninensis* to discriminate closely related congeneric species of *Rubus*. Taken together, the results of this comparative plastome study will shed new light on chloroplast genome structure and evolution of insular endemic species pairs during anagenetic speciation and contribute to the development of chloroplast markers based on mutation hotspot regions, thereby facilitating resolution of phylogenetic relationships among closely congeneric species of *Rubus*.

## 2. Materials and Methods 

### 2.1. Plastome Sequencing and Annotation

Fresh leaves of a single plant of *R. boninensis* were collected from the Volcano Islands group in the Bonin Islands (i.e., Minamiiwojima), Japan (voucher specimen: KYO_Takayama17062202). Similarly, leaves of *R. trifidus* were collected from Yigidae, Busan, southern part of Korea peninsula (voucher specimen: KNU_Yigidae180513) and dried with silica gel before DNA extraction. Total DNA was isolated by using the DNeasy Plant Mini Kit (Qiagen, Carlsbad, CA, USA) and sequenced with an Illumina HiSeq 4000 (Illumina, Inc., San Diego, CA, USA), yielding 150 bp paired-end read length, at Macrogen Corporation (Seoul, Korea). A total of 22,273,138 and 43,891,068 paired-end reads were obtained for *R. boninensis* and *R. trifidus*, respectively, and assembled de novo using Velvet v. 1.2.10 with multiple k-mers [23]. The tRNAs were confirmed using with tRNAscan-SE [24]. Annotation was conducted using Geneious R10 [25] and the annotated plastome sequences were submitted to GenBank (accession numbers MH734123 and MK465682 for *R. boninensis* and *R. trifidus*, respectively). The annotated GenBank format sequence file was used to draw a circular map with OGDRAW program v1.2 [26].

### 2.2. Comparative Plastome Analysis

The complete plastomes of *R. boninensis* and *R. trifidus* were compared to those of two other *Rubus* species, *R. crataegifolius* (MG189543) and *R. takesimensis* (MH734123), using mVISTA [27] in Shuffle-LAGAN mode [28]. The four *Rubus* plastome sequences were aligned with MAFFT v. 7 [29] and adjusted manually with Geneious [25]. By using DnaSP v. 6.10 software [30], a sliding window analysis with a step size of 200 bp and window length of 800 bp was carried out to determine the nucleotide diversity (*Pi*) of the plastome. The codon usage frequency was calculated using MEGA7 [31] with relative synonymous codon usage (RSCU) value [32], which is a simple measure of non-uniform usage of synonymous codons in a coding sequence. The DNA code used by bacteria, archaea, prokaryotic viruses, and chloroplast proteins was used [33].

### 2.3. Tandem Repeat and Microsatellite Analysis

Microsatellite or SSR markers were identified in the plastome sequences by using MISA [34] with minimum repeat thresholds of ten for mononucleotide repeats, four for dinucleotide repeats, four for trinucleotide repeats, four for tetranucleotide repeats, four for pentanucleotide repeats, and three for hexanucleotide repeats [22]. 

### 2.4. Phylogenetic Analysis

For the phylogenetic analysis, the complete plastome sequences of 18 representative species from the family Rosaceae (seven species from *Rubus*, including *R. corchorifolius* (KY419958), *R. niveus* (KY419961), and *R. fockeanus* (KY420018); six species from *Fragaria*; two species from *Rosa*; one species from *Prunus*; two species from *Pyrus*; and one species from *Prinsepia*) were aligned with MAFFT v. 7 [29] in Geneious [25]. Maximum likelihood (ML) analysis based on the best-fit model of TVM+F+R2 was conducted with IQ-TREE v. 1.4.2 [35]. *Prinsepia utilis* was used as an outgroup, and non-parametric bootstrap analysis was performed with 1000 replicates.

## 3. Results and Discussion

### 3.1. Genome Size and Features

**Figure 1:**
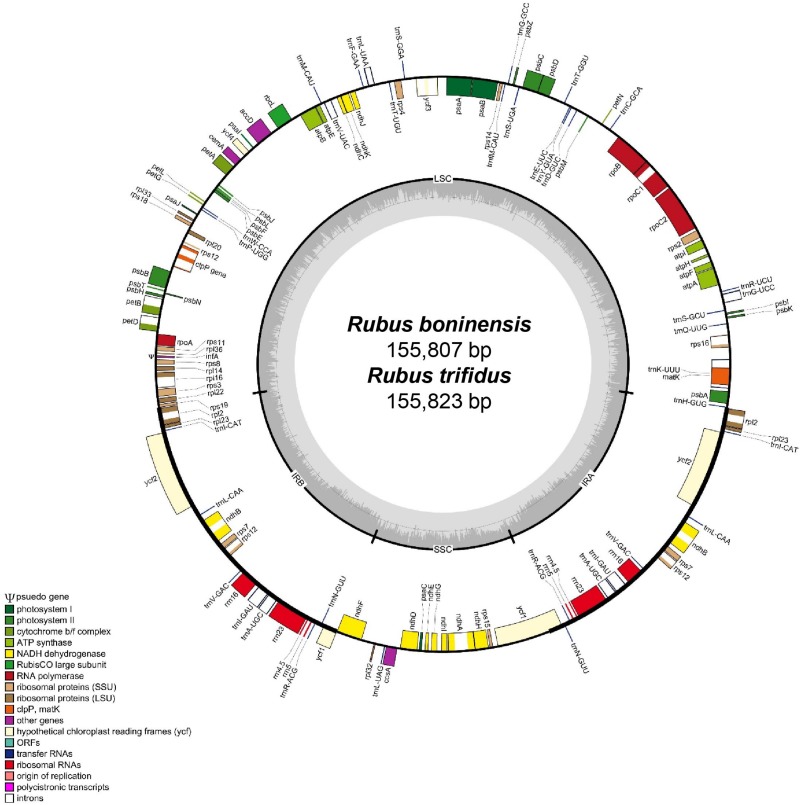
The complete plastome map of *R. boninensis* and *R. trifidus*. The genes located outside of the circle are transcribed clockwise, while those located inside are transcribed counterclockwise. The gray bar area in the inner circle denotes the guanine-cytosine (GC) content of the genome, whereas the lighter gray area indicates the adenosine-thymine (AT) content of the genome. Large single copy, small single copy, and inverted repeat are indicated with LSC, SSC, and IR, respectively. Ψ indicates pseudogenes.

The complete plastome sequence of *R. boninensis* was 155,807 bp long, with a large single copy (LSC) region of 85,438 bp, small single copy (SSC) region of 18,783 bp, and two inverted repeat (IR) regions of 25,793 bp. The *R. trifidus* plastome was 155,823 bp long, with a large single copy (LSC) region of 85,466 bp, small single copy (SSC) region of 18,759 bp, and two inverted repeat (IR) regions of 25,799 bp (Figure 1 and Table 1). The two plastomes of *R. boninensis* and *R. trifidus* contained 131 genes, including 84 protein-coding, 8 ribosomal RNA, and 37 transfer RNA genes. The overall guanine-cytosine (GC) content of both *R. boninensis* and *R. trifidus* was 37.1%. 

In both the species, 17 genes were duplicated in the IR regions, including seven tRNA, four rRNA, and six protein-coding genes. Fifteen genes (*ndhA, ndhB, petB, petD, rpl2, rpl16, rpoC1, rps12, rps16, trnA-UGC, trnG-UCC, trnI-GAU, trnK-UUU, trnL-UAA*, and *trnV*-*UAC*) contained one intron, whereas *clpP* and *ycf3* each contained two introns. Interestingly, the highly conserved group II intron of *atpF* was lost, as we have demonstrated in the case of *R. crataegifolius* and *R. takesimensis* [22]. It remains to be determined if loss of the *atpF* intron, which occurs frequently in the two genera, *Rosa* and *Rubus*, has also occurred in the other major lineages of Rosaceae and related Rosid families [22,36]. A partial *ycf1* gene (1221 bp in both the species, *R. boninensis* and *R. trifidus*) was located at the IR_b_/SSC junction region, whereas the complete *ycf1* gene was located in the IR region at the SSC/IR_a_ junction. To reveal a hybrid origin of some endemic taxa of *Rubus* (subgenus *Idaeobatus*) in the Hawaiian Islands, Howarth et al., [37] successfully used the *ndhF* gene, which is known to have frameshift mutations and alterations on transcription termination due to the higher substitution rates, a wide range of insertion and deletion (indel) variations, and a high AT content [38]. Although the closely related *Rosa* section *Synstylae* showed frameshift mutations on the 3’ end of the *ndhF* gene [39], only nucleotide substitution and alteration on transcription were found in the four species of *Rubus*, without size variation (a total CDS length of 2244, which is the same as that of *Rosa* section *Synstylae*). The *infA* gene, which was located in the LSC region, became a pseudogene. The plastome sequence of the insular derived species, *R. boninensis*, was highly similar to that of the continental progenitor species, *R. trifidus* (99.6% sequence similarity; 155,355 bp identical sites), and the *R. trifidus* plastome sequence was just 16 bp longer than that of *R. boninensis* (Table 1). In case of the species pair in East Sea/Sea of Japan, the complete plastome sequences of *R. takesimensis* and *R. crataegifolius* were 99.8% similar (i.e., 155,537 bp identical sites); the *R. takesimensis* plastome was 46 bp longer than the *R. crataegifolius* plastome (a 28 bp extension in the LSC and a 18 bp extension in the SSC) [22].

The frequency of codon usage in *R. boninensis* and *R. trifidus* was calculated for their plastomes based on protein-coding genes and tRNA genes (Table 2). The codon usage bias (CUB) refers to differences in the frequency of occurrence of synonymous codons in coding DNA, and it has been demonstrated that CUBs could be manifested by maintaining a balance between mutational bias and natural selective forces [40,41]. Therefore, analysis and characterization of CUBs at the genomic scale can help elucidate molecular evolution and environmental adaptation [42]. Overall, we detected similar patterns in codon usage between *R. boninensis* and *R. trifidus*. Some exceptions included AUG codon usage of *trnI-CAU*, *trnfM-CAU*, and CAA codon usage of *trnK-UUG* in *R. boninensis*; and UAG and UCA codon usage of *trnI-CAT* and *trn*S*-UGA*, respectively, in *R. trifidus*. The frequency of codon usage of *R. crataegifolius* and *R. takesimensis* is also summarized in Table 3. When compared with the pair of *R. boninensis*–*R. trifidus*, AUG (*trnI-CAU, trnfM-CAU*, and *trnM-CAU*), UCA (*trn*S*-UGA*), UAG (no usage), and CAA codon usage (*trnQ-UUG*) showed different patterns. The codon usage of two pairs of *Rubus* species (*R. boninensis*–*R. trifidus* and *R. crataegifolius*–*R. takesimensis*) was biased toward a high RSCU values of U and A at the third codon usage, a similar phenomenon found in other angiosperm [43] and algal lineages [40]. 

### 3.2. Analysis of Microsatellites

**Figure 2:**
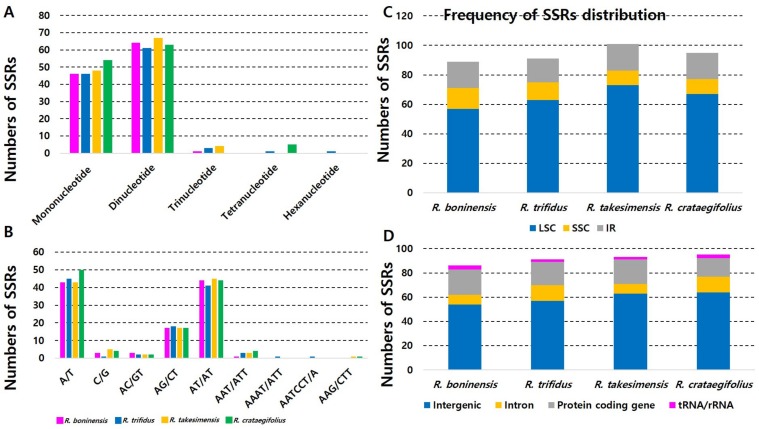
Analyses of repeated sequences in four *Rubus* plastome sequences. (**A**) Numbers of three repeat types; (**B**) numbers of identified SSRs motifs in different repeat class types; (**C**) frequency of repeat types in LSC, SSC, and IR regions; (**D**) frequency of repeat types in intergenic, intron, protein coding region, and tRNA/rRNA.

We found a nearly identical number of potential SSRs between the continental progenitor, *R. trifidus* (a total of 112 SSRs), and insular derived, *R. boninensis*, in the Bonin Islands (a total of 111 SSRs). Of a total of 86 unique consensus sequences (out of 111 copies) identified in *R. boninensis*, 57 (66.3%) were located in the LSC region, 11 in the SSC region (12.8%), and 18 (20.9%) in the two IR regions (Supplementary Table S1). Of a total of 91 unique consensus sequences (out of 112 copies) identified in *R. trifidus*, 63 (69.2%) were located in the LSC region, 12 in the SSC region (13.2%), and 16 (17.6%) in the two IR regions (Supplementary Table S2). Therefore, we found slight differences in the number of SSRs obtained between *R. boninensis* and *R. trifidus*. In addition, mononucleotide repeats were detected in 46 (41.4%) and 46 (41.1%) SSRs in *R. boninensis* and *R. trifidus*, respectively, while very low frequencies of 1 (0.9%) and 3 (2.7%) for trinucleotide repeats were found in *R. boninensis* and *R. trifidus*, respectively (Figure 2A).

No tetranucleotides, pentanucleotides, and hexanucleotides were found in *R. boninensis*; however, one tetranucleotide and one hexanucleotide repeat were identified in *R. trifidus*. The most common SSR motifs in the *R. boninensis* and *R. trifidus* plastomes were dinucleotide repeats; 64 (57.7%) and 61 (54.5%), respectively (Figure 2A). Regarding the location of SSRs, they were located in intergenic regions (54 (62.8%) and 57 (62.6%) in *R. boninensis* and *R. trifidus*, respectively), 8 (9.3%; *R. boninensis*) and 13 (14.3%; *R. trifidus*) in introns, and 21 (24.4%; *R. boninensis*) and 19 (20.9%; *R. trifidus*) in protein coding genes (Figure 2D). Besides, three SSRs (3.5%) were located in tRNA (*trnS-UGA*) and rRNA (23S rRNA) of *R. boninensis*, and two (2.2%) were located in 23S rRNA genes of *R. trifidus*. Moreover, 72.1% and 76.9% of the SSRs were located in intergenic and intron regions, respectively, whereas only 27.9% and 23.1% were distributed in the conserved gene regions of *R. boninensis* and *R. trifidus*, respectively (Figure 2D). To compare with previously reported SSRs of *R. takesimensis* of endemic species on Ulleung Island in East Sea/Sea of Japan, we also analyzed SSRs of *R. crataegifolius*, the continental progenitor, and found a slightly higher number of potential SSRs (a total of 122) for *R. crataegifolius* (Supplementary Table S3). Of a total of 95 unique consensus sequences (out of 122 copies) identified in *R. crataegifolius*, 67 (70.5%) were located in the LSC region, 10 in the SSC region (10.5%), and 18 (19%) in the two IR regions (Figure 2C). In addition, mononucleotide and trinucleotide repeats were detected in 54 (44.3%) and 5 (4.1%) SSRs, respectively (Figure 2A). The most common SSR motifs in the *R. crataegifolius* plastome were dinucleotide repeats (a total of 63; 51.6%); however, no tetranucleotides, pentanucleotides, and hexanucleotides were found (Figure 2A). Like other congeneric species, most of the SSRs (64 and 67.4%) were located in intergenic regions, while 13 (13.7%) were located in introns and 15 (15.8%) in protein coding genes. In addition, three (3.1%) SSRs were located in tRNA (*trnS-UGA*) and rRNA (23S rRNA) genes. When the insular derived species, *R. takesimensis*, and the continental progenitor, *R. crataegifolius*, were compared, *R. crataegifolius* showed a slightly higher number of SSRs in the intergenic region (64; 67.4%) and slightly lower number of SSRs in the intron region (13; 13.7%) (Figure 2D). The other characteristics of SSRs found in *R. crataegifolius* were similar to those of *R. takesimensis*. The pair of continental progenitor- and insular-derived species in the Bonin Island of the northwestern Pacific Ocean showed nearly identical numbers of SSRs between *R. boninensis* (a total of 111) and *R. trifidus* (a total of 112), with similar percentages located in the LSC region (*R. boninensis* and *R. trifidus*, 66.3% and 69.2%, respectively). Also, the insular-derived species of the Bonin and Ulleung Islands showed a slightly higher number of potential SSRs in the IR region (*R. boninensis* with 18 (20.9%) and *R. takesimensis* with 20 (19.8%)) than that of the continental progenitors (*R. trifidus* with 16 (17.6%) and *R. crataegifolius* with 18 (19.4%)). Lastly, the protein coding genes also showed similar patterns: *R. boninensis* with 21 (24.4%) and *R. takesimensis* with 20 (19.8%) versus *R. trifidus* with 19 (20.9%) and *R. crataegifolius* with 15 (15.8%) (Figure 2).

Compared to the three recently reported plastomes of *Rosa* section *Synstylae* [39] and other members of Rosoideae (i.e., *Fragaria* × *ananassa* [44], *Rosa chinensis* var. *spontanea* [45]), with similar motif search parameter settings, the total numbers of SSR motifs found in the four *Rubus* species (*R. boninensis* (*n* = 111) and *R. trifidus* (*n* = 112) in subgenus *Anoplobatus*; *R. crataegifolius* (*n* = 122) and *R. takesimensis* (*n* = 116) in subgenus *Idaeobatus*) were significantly higher than that in *Rosa* section *Synstylae* (*n* = 87), *Fragaria* × *ananassa* (*n* = 61), and *Rosa chinensis* var. *spontanea* (*n* = 58). Furthermore, the number of dinucleotide repeats was significantly higher in the four *Rubus* species (51.6%–57.8%; *n* = 61–67) than in *Rosa* section *Synstylae* (12.6%; *n* = 11) and *Rosa chinensis* var. *spontanea* (11.8%; *n* = 10). Also, the highest proportions of mononucleotides motifs were found in nine species of *Malus* chloroplast genomes, with the total number of chloroplast SSR (cpSSRs) ranging from 94 to 101 [46]. Therefore, it is yet to be ascertained whether the conserved SSR motifs found in the four *Rubus* species of subgenus *Anoplobatus* and *Idaeobatus* in this study can also be found in other major lineages of *Rubus*, such as subgenus *Malachobatus* and *Rubus*. The locations of SSR motifs and A/T abundance found in *R. boninensis*, *R. trifidus*, and *R. crataegifolius* (Figure 2B) were consistent with other members of Rosaceae [39,44,45,46,47]. As the utility of cpSSRs has been proven to be valuable in various plant lineages (e.g., *Glycine*, [48]; *Pinus*, [49]; *Triticum*, [50]; *Abies*, [51]; *Cucumis*, [52]), we believe that the cpSSR markers developed in this study can be useful for complex studies at both the population and specific level of members of subgenus *Anoplobatus* and *Idaeobatus*.

### 3.3. Comparative Analysis of Genome Structure 

**Figure 3:**
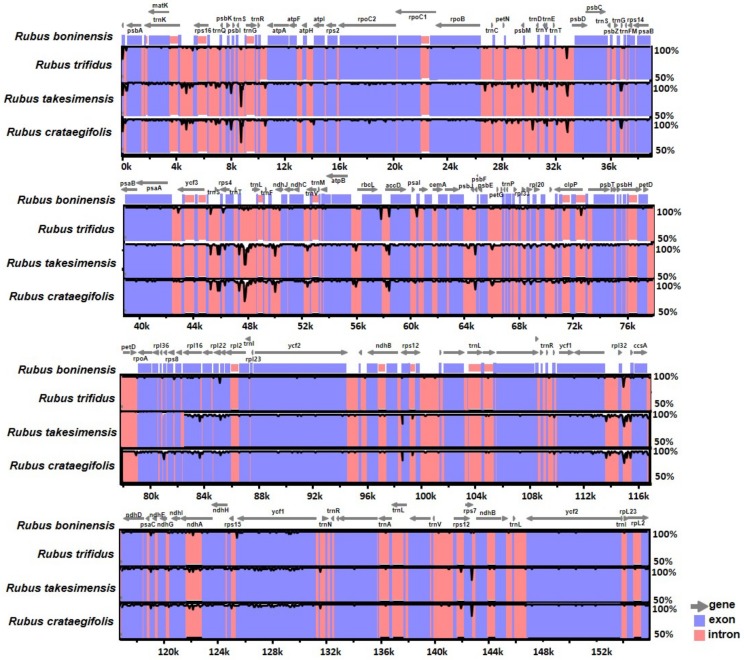
Visualization of alignment of four *Rubus* species’ chloroplast genome sequences. The VISTA-based identity plots show the sequence identity of four chloroplast genomes with reference to *R. boninensis*. Vertical scale indicates the percent identity from 50% to 100%. Coding and non-coding regions are in blue and pink, respectively. Gray arrows above the alignment indicate the position and direction of each gene.

The complete plastome sequences of *R. boninensis*, *R. trifidus*, *R. takesimensis*, and *R. crataegifolius* were plotted by using mVISTA analysis, based on the annotated *R. boninensis* plastome as a reference (Figure 3). As expected, we found that the LSC region was the most divergent and the two IR regions were highly conserved, and also that the non-coding regions were more divergent and variable than the coding regions. In addition, the *R. boninensis* plastome was most similar (i.e., 99.6% sequence similarity; 155,355 bp identical sites) to the *R. trifidus* plastome, which belongs to the same subgenus, *Anoplobatus*, and least similar (98.6% sequence similarity; 154,123 bp identical sites) to the plastome of *R. takesimensis*, which belongs to a different subgenus, *Idaeobatus*.

**Figure 4:**
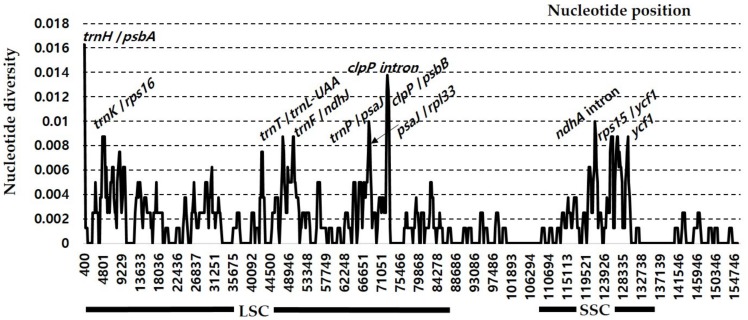
Sliding window analysis of the whole chloroplast genomes of *R. boninensis* and *R. trifidus*.

**Figure 5:**
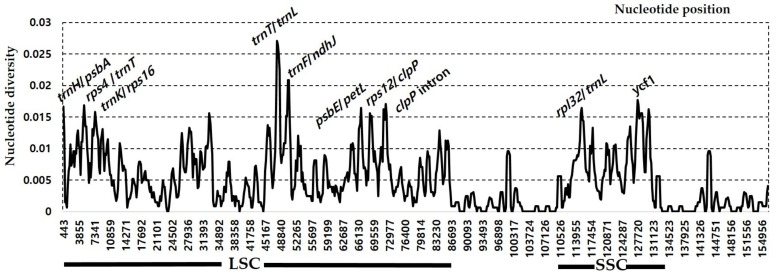
Sliding window analysis of the whole chloroplast genomes of four *Rubus* species (*R. boninensis*, *R. trifidus, R. crataegifolius*, and *R. takesimensis*).

The sliding window analysis conducted by using DnaSP revealed highly variable regions in the insular endemic taxa and progenitor pairs of *Rubus* chloroplast genome (Figure 4). When the insular derivative, *R. boninensis*, and the continental progenitor, *R. trifidus*, were compared, the average value of nucleotide diversity (*Pi*) over the entire cp genome was 0.002. The most variable region was the *trnH*/*psbA* intergenic region with a *Pi* value of 0.016. Also, the highly variable regions included ten other intergenic regions, i.e., *clpP intron*1 (*Pi* = 0.01375), *clpP/psbB* (*Pi* = 0.01125), *trnP/psaJ* (*Pi* = 0.01), *ndhA* intron (*Pi* = 0.01), *trnK/rps16* (*Pi* = 0.00875), *trnT/trnL-UAA* (*Pi* = 0.00875), *trnF/ndhJ* (*Pi* = 0.00875), *psaJ/rpl33* (*Pi* = 0.00875), *rps15/ycf1* (*Pi* = 0.00875), and one genic region *ycf1* (*Pi* = 0.00875). Therefore, these 11 regions, including *trnH/psbA*, will be good candidate organelle markers to make phylogenetic inference and carry out phylogeographic studies of *Rubus* subgenus *Anoplobatus*. In the four *Rubus* plastid genomes including species pair (*R. crataegifolius* and *R. takesimensis*) in subgenus *Idaeobatus*, we found more than two times (i.e., 0.005) the average nucleotide diversity (*Pi*) over the entire cp genome compared to that in the species pair (*R. boninensis* and *R. trifidus*) in subgenus *Anoplobatus*. The most variable region in the four *Rubus* plastomes was the *trnT/trnL* intergenic region with a *Pi* value of 0.027, which was much higher than that in the *R. boninensis*–*R. trifidus* species pair (Figure 5). Also, highly variable regions included seven other intergenic regions, i.e., *trnF/ndhJ* (*Pi* = 0.02083), *trnK/rps16* (*Pi* =0.01688), *psbE/petL* (*Pi* = 0.01646), *rpl32/trnL* (*Pi* = 0.01646), *trnH/psbA* (*Pi* = 0.01667), *rps*4*/trnT* (*Pi* = 0.01667), and *rps12/clpP* (*Pi* = 0.01625), one intron region *clpP intron*2 (*Pi* = 0.01708), and one genic region *ycf1* (*Pi* = 0.01771). Thus, a total 10 highly variable regions with *Pi* values of greater than 0.016 were identified in the four *Rubus* plastid genomes (Figure 5). Based on the above results, the nine variable regions including *trnT/trnL*, may serve to resolve phylogenetic relationships between subgenus *Anoplobatus* and subgenus *Idaeodobatus*. Our earlier study [22] identified six hotspot regions in the comparative analysis of *Rubus* subgenus *Idaeobatus* and subgenus *Cylactis*-*trnL/trnF, rps16/trnQ, ndhD/psaC, trnK/rps16, trnQ/psbK*, and *trnM/psaC* with high *Pi* values > 0.03. When a previous study [22] on *Rubus* for the identification of variable regions across the complete chloroplast genome is considered, the *trnK/rps16* intergenic region was a common hotspot region within genus *Rubus* (subgenus *Anoplobatus*, *Idaeobatus*, and *Cylactis*). Although two genic (*psbA* and *atpA*) and five intergenic (*trnQ/rps16, ndhC/trnV, trnR/atpA, ndhF/rpl32*, and *psbM/trnD*) hotspot regions were reported in *Prunus* [53] and *Pyrus* [54], respectively; three other conventional barcoding genic regions (i.e., *rbcL, matK*, and *rpoC1*) have been used widely as standard markers for species identification in Rosaceae [55]. Based on three *Pyrus* chloroplast genome sequences, four hotspot regions (i.e., *petN-psbM, psbM-trnD, rps4-trnT-trnL*, and *psaI-ycf4*) with an average *Pi* value as 0.00054 were suggested as effective makers for phylogeny and conservation genetics in the genus *Pyrus* [56]. In nine *Malus* chloroplast genomes, some divergence in intergenic spacer and introns was found, including *trnK/rps16, trnT/trnL*, and *clpP* introns and coding regions *(matK, rpoA, ndhF*, and *ycf1*) [46]. In *Rosa* section *Synstylae*, closely related taxa in the same subfamily Rosoideae as *Rubus*, Jeon and Kim [39] found six highly variable regions with *Pi* value of >0.006: *psbI-trnS-trnG*, 5’*matK-trnK*, *rps16-trnG, rpoB-trnC, rps4-trnT*, and *ycf1*. It is interesting to notice that, compared to the *Pi* value of four *Rubus* species, *Rosa* species showed substantially lower nucleotide diversity, with the highest *Pi* value of 0.01313 and very few mutations in their chloroplast genomes. Also, we found that *clpP* intron, *rps4/trnT*, *trnK/rps16, trnT/trnL*, and *ycf1* represent common hotspots in both *Rubus* and other Rosaceae. Therefore, in summary, we identified several highly variable plastome regions within genus *Rubus* subgenus *Anoplobatus* (*clpP/psbB, trnP/psaJ, ndhA intron, trnF/ndhJ, psaJ/rpl33*, and *rps15/ycf1*) and genus *Rubus* (*trnF/ndhJ, psbE/petL, rpl32/trnL*, and *rps12/clpP*). In conjunction with nuclear markers, these highly variable regions, as effective maternally inherited markers, can be applied to explore the highly complex evolutionary history of these groups.

### 3.4. Phylogenetic Analysis

**Figure 6:**
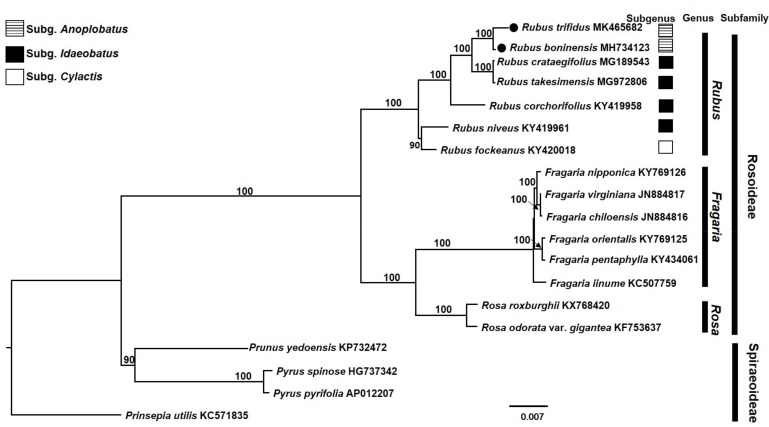
The maximum-likelihood (ML) tree inferred from 19 representative species of Rosaceae. The bootstrap value based on 1000 replicates is shown for each node.

Maximum likelihood analysis of complete cp genome sequences, which included 19 representative members of the family Rosaceae, was carried out based on the best-fit model of TVM+F+R2. Of a total of 170,274 aligned nucleotide bases, 149,000 (87.5%) were constant and 21,274 (12.5%) were variable sites, with 8273 (4.9%) parsimony-informative sites. The ML tree supported the monophyly of *Rubus* and the sister relationship between the continental progenitor, *R. trifidus*, and *R. boninensis* in the Bonin Islands of the northwestern Pacific Ocean in subgenus *Anoplobatus*, and also between the continental progenitor, *R. crataegifolius*, and the insular derivative, *R. takesimensis* in the East Sea/Sea of Japan in subgenus *Idaeobatus* (Figure 6). The complete chloroplast genome sequences provided full resolutions within *Rubus*, with high support values (all but one 100% BS support). The ML tree also provided evidence that the currently delimited subgenus *Idaeobatus* is not monophyletic. Given the lack of sufficient resolution and insufficient support for relationships of interest within *Rubus* [57,58,59,60,61], phylogenomic study (or phylogenetic study based on hotspot regions identified in this study) within the genus will shed new light on the disentangling of complex evolutionary events within the genus. Furthermore, inferences based on large-scale phylogenetic frameworks and our understanding of trait evolution within Rosaceae should benefit from phylogenomic approaches based upon whole-plastome sequencing [62,63].

## 4. Conclusions

The complete plastome sequences of the insular derived *R. boninensis*, a rare plant endemic to the Bonin Islands in the northwestern Pacific Ocean and *R. trifidus*, a continental progenitor, were determined. Their plastome sequences were compared to those of *R. crataegifolius*, the continental progenitor and *R. takesimensis*, the insular derivative on Ulleung Island in East Sea/Sea of Japan. These species pairs represent parallel plastome systems in two different subgenera of *Rubus*, *Anoplobatus* and *Idaeobatus*, providing insights into plastid genome evolution during anagenetic speciation. Both *infA* pseudogenization and *atpF* intron loss were observed in the two species pairs. The relative synonymous codon usage of genus *Rubus* was biased toward high RSCU values of U and A at the third codon. Several mutation hotspot regions for the *R. boninensis*–*R. trifidus* species pair included *trnH/psbA, clpP* intron1, *clpP/psbB, trnP/psaJ, ndhA* intron, *trnK/rps16, trnT/trnL, trnF/ndhJ, psaJ/rpl33, rps15/ycf1*, and *ycf1*. Based on four complete plastome sequences of *Rubus*, ten highly variable regions, including *trnT/trnL, trnF/ndhJ, clpP* intron2, *trnK/rps16, psbE/petL*, *rpl32/trnL, trnH/psbA, rps4/trnT, rps12/clpP*, and *ycf1* were detected. The application of these markers will be a powerful tool for barcoding and for cultivar and germplasm identifications for economically important *Rubus* and related genera in Rosaceae. In addition to the identification of hotspot regions, we also identified cpSSRs for our four species of interest. We found a higher number of SSRs for the continental progenitor species *R. crataegifolius* and *R. trifidus* than for the insular derived species *R. boninensis* and *R. takesimensis*. The most common SSR motifs in the four *Rubus* species were dinucleotide repeats; however, such repeats were not found in other Rosaceae genera (e.g., *Malus* and *Rosa*). The location of SSRs motifs and A/T abundance detected from four *Rubus* species were consistent with other members of Rosaceae. The phylogenetic analysis confirmed the evolution of two anagenetically derived insular species from their continental progenitors. Additional studies using multiple samples from continental and island species based on highly variable plastome markers found in this study can ultimately confirm such progenitor–derivative relationships and help us better understand the anagenetic speciation of island endemics. In addition, the phylogenomic analysis of complete plastome sequences will be an effective tool to infer phylogenetic relationships within *Rubus* and to establish infra-familial classifications within Rosaceae.

## Figures and Tables

**Table 1:** Summary of the characteristics of *R. boninensis* and *R. trifidus* chloroplast genomes.

Taxa	*R. boninensis*	*R. trifidus*
Accession Number	MH734123	MK465682
Total cpDNA size (bp)/GC content (%)	155,807/37.1	155,823/37.1
LSC size (bp)/GC content (%)	85,438/34.9	85,466/35.0
IR size (bp)/GC content (%)	25,793/42.8	25,799/42.8
SSC size (bp)/GC content (%)	18,783/31.0	18,759/31.0
Number of genes	131	131
Number of protein-coding genes	84	84
Number of tRNA genes	37	37
Number of rRNA genes	8	8
Number of duplicated genes	17	17

LSC: Large single copy region, IR: Inverted repeat, SSC: Small single copy region.

**Table 2:** Codon usage and codon–anticodon recognition pattern for tRNA in *R. boninensis* and *R. trifidus* cp genome. Species abbreviations, BON and TRF, represent *R. boninensis* and *R. trifidus*, respectively.

Codon	Amino Acid	Count BON/TRF	RSCU BON/TRF	tRNA BON/TRF	Codon	Amino Acid	Count BON/TRF	RSCU BON/TRF	tRNA BON/TRF
UUU	F	986/985	1.31		UCU	S	561/554	1.68/1.67	
UUC	F	514	0.69	*trnF-GAA*	UCC	S	343	1.03	*trnS-GGA*
UUA	L	882/887	1.92/1.93	*trnL-UAA*	UCA	S	382/380	1.15	*- / trnS-UGA*
UUG	L	550/552	1.2	*trnL-CAA*	UCG	S	200/204	0.6	
CUU	L	592/590	1.29		CCU	P	408/409	1.5	
CUC	L	185/138	0.4		CCC	P	212/213	0.78	
CUA	L	356/355	0.78/0.77	*trnL-UAG*	CCA	P	304/300	1.12/1.1	*trnP-UGG*
CUG	L	185/186	0.4/0.41		CCG	P	164	0.6	
AUU	I	1118/1114	1.48		ACU	T	523/525	1.57	
AUC	I	435	0.58	*trnI-GAU*	ACC	T	254	0.76/0.78	*trnT-GGU*
AUA	I	711/703	0.94		ACA	T	406/407	1.22	*trnT-UGU*
**AUG**	M	619/620	1	*trnI-CAU, trnfM-CAU/trnfM-CAU*	ACG	T	148	0.44	
GUU	V	518	1.45		GCU	A	630	1.8/1.81	
GUC	V	169/167	0.47	*trnV-GAC*	GCC	A	219	0.63	
GUA	V	543	1.52	*trnV-UAC*	GCA	A	387/384	1.11	*trnA-UGC*
GUG	V	202/201	0.56		GCG	A	162	0.46	
UAU	Y	768/767	1.59		UGU	C	229	1.53	
UAC	Y	196	0.41	*trnY-GUA*	UGC	C	71	0.47	*trnC-GCA*
UAA	*	48	1.71/1.73		UGA	*	16	0.57/0.58	
**UAG**	*	20	0.71/0.69	*-/trnI-CAT*	UGG	W	454/452	1	*trnW-CCA*
CAU	H	486/487	1.53		CGU	R	343/344	1.3/1.31	*trnR-ACG*
CAC	H	151/150	0.47	*trnH-GUG*	CGC	R	104	0.39	
**CAA**	Q	711/713	1.54/1.55	*trnK-UUG -*	CGA	R	357/358	1.36	
CAG	Q	210/209	0.46/0.45		CGG	R	116/115	0.44	
AAU	N	971/968	1.52/1.51		AGU	S	390/387	1.17/1.16	
AAC	N	310/311	0.48/0.49	*trnN-GUU*	AGC	S	125/126	0.37/0.38	*trnS-GCU*
AAA	K	1083/1070	1.5/1.49	*trnK-UUU*	AGA	R	482	1.83	*trnR-UCU*
AAG	K	362	0.5/0.51		AGG	R	178	0.68	
GAU	D	873/874	1.62		GGU	G	581/582	1.3	
GAC	D	203/202	0.38	*trnD-GUC*	GGC	G	194/193	0.43	*trnS-GCC*
GAA	E	1020/1022	1.48/1.49	*trnE-UUC*	GGA	G	701/699	1.57	*trnG-UCC*
GAG	E	358/354	0.52/0.51		GGG	G	313/312	0.7	

Asterisk (*) denotes stop codon. RSCU: Relative synonymous codon usage. Bold type denotes the exception of codon usage.

**Table 3:** Codon usage and codon–anticodon recognition pattern for tRNA in *R. crataegifolius* and *R. takesimensis* cp genome. Species abbreviations, CRA and TAK, represent *R. crataegifolius* and *R. takesimensis*, respectively.

Codon	Amino Acid	Count CRA/TAK	RSCU CRA/TAK	tRNA CRA/TAK	Codon	Amino Acid	Count CRA/TAK	RSCU CRA/TAK	tRNA CRA/TAK
UUU	F	980/979	1.31		UCU	S	557	1.68	
UUC	F	514	0.69	*trnF-GAA*	UCC	S	345	1.04	*trnS-GGA*
UUA	L	886/887	1.93	*trnL-UAA*	UCA	S	383	1.15	*trnS-UGA*
UUG	L	557/555	1.21	*trnL-CAA*	UCG	S	198	0.6	
CUU	L	593/592	1.29		CCU	P	412	1.52	
CUC	L	181	0.39		CCC	P	211	0.78	
CUA	L	363/361	0.79	*trnL-UAG*	CCA	P	303	1.11	*trnP-UGG*
CUG	L	181/182	0.39/0.4		CCG	P	161	0.59	
AUU	I	1105/1105	1.48		ACU	T	525/523	1.57	
AUC	I	442	0.59	*trnI-GAU*	ACC	T	251	0.75	*trnT-GGU*
AUA	I	693	0.93		ACA	T	406/408	1.22	*trnT-UGU*
**AUG**	M	617	1	*trnI-CAU, trnfM-CAU, trnM-CAU*	ACG	T	152/151	0.46/0.45	
GUU	V	519	1.45		GCU	A	634	1.82	
GUC	V	172	0.48	*trnV-GAC*	GCC	A	218	0.63	
GUA	V	536/538	1.5/1.51	*trnV-UAC*	GCA	A	377/376	1.08	*trnA-UGC*
GUG	V	201/200	0.56		GCG	A	163/164	0.47	
UAU	Y	766	1.59		UGU	C	229	1.53	
UAC	Y	195	0.41	*trnY-GUA*	UGC	C	70	0.47	*trnC-GCA*
UAA	*	47	1.68		UGA	*	14	0.5	
**UAG**	*	23	0.82		UGG	W	451	1	*trnW-CCA*
CAU	H	479/477	1.52/1.51		CGU	R	341	1.29	*trnR-ACG*
CAC	H	152/154	0.48/0.49	*trnH-GUG*	CGC	R	105/104	0.49/0.39	
**CAA**	Q	714	1.54	*trnQ-UUG*	CGA	R	362/360	1.36	
CAG	Q	211	0.46		CGG	R	116/117	0.44	
AAU	N	969	1.52		AGU	S	388/387	1.17/1.16	
AAC	N	307	0.48	*trnN-GUU*	AGC	S	124/125	0.37	*trnS-GCU*
AAA	K	1065/1066	1.5	*trnK-UUU*	AGA	R	486/488	1.83/1.84	*trnR-UCU*
AAG	K	353/352	0.5		AGG	R	182	0.69	
GAU	D	867	1.62		GGU	G	576/577	1.29	
GAC	D	204/205	0.38	*trnD-GUC*	GGC	G	198/197	0.44	*trnS-GCC*
GAA	E	1017	1.48	*trnE-UUC*	GGA	G	700/697	1.57	*trnG-UCC*
GAG	E	358	0.52		GGG	G	311/313	0.7	

Asterisk (*) denotes stop codon. RSCU: Relative synonymous codon usage. Bold type denotes the exception of codon usage.

## Supplementary Materials

The following are available online at https://www.mdpi.com/2073-4425/10/10/774/s1, Table S1: Distribution, length, and location of repeat sequences in the plastome sequence of *Rubus boninensis* in the Bonin Islands, Table S2: Distribution, length, and location of repeat sequences in the complete plastome sequence of *Rubus trifidus* from the southern Korean peninsula, Table S3: Distribution, length, and location of repeat sequences in the *Rubus crataegifolius* plastome sequence.
