# Supplementary material for: Comparison of the Whole-Plastome Sequence between the Bonin Islands Endemic Rubus boninensis and Its Close Relative, Rubus trifidus (Rosaceae), in the Southern Korean Peninsula
Source: Genes (Basel). 2019 Oct 2;10(10):774. doi: 10.3390/genes10100774 (PMC6826710; doi:10.3390/genes10100774)
Supplement: Supplementary file 1 [file genes-10-00774-s001.zip › Table S1.docx]

**Table S1.** Distribution, length, and location of repeat sequences in the *Rubus boninensis* plastome sequence.

| cpSSR ID | Repeat Motif | Length (bp) | Start | End | Region | Annotation |
| --- | --- | --- | --- | --- | --- | --- |
| 1 | (TA)4 | 8 | 1620 | 1627 | LSC |  |
| 2 | (T)11 | 11 | 2196 | 2206 | LSC | *matK* |
| 3 | (A)10 | 10 | 4294 | 4303 | LSC |  |
| 4 | (T)10 | 10 | 4462 | 4471 | LSC |  |
| 5 | (TA)4 | 8 | 4770 | 4777 | LSC |  |
| 6 | (AG)5 | 10 | 6803 | 6812 | LSC |  |
| 7 | (AT)4 | 71 | 7155 | 7225 | LSC |  |
| 8 | (A)10 | 10 | 7925 | 7934 | LSC |  |
| 9 | (A)11 | 11 | 8684 | 8694 | LSC |  |
| 10 | (T)13 | 13 | 12248 | 12260 | LSC |  |
| 11 | (C)10 | 20 | 13952 | 13971 | LSC |  |
| 12 | (T)11 | 11 | 18063 | 18073 | LSC | *rpoC2* |
| 13 | (TA)5 | 10 | 19417 | 19426 | LSC | *rpoC2* |
| 14 | (T)10 | 10 | 25764 | 25773 | LSC | *rpoB* |
| 15 | (AT)4 | 8 | 26163 | 26170 | LSC | *rpoB* |
| 16 | (T)10 | 10 | 28297 | 28306 | LSC |  |
| 17 | (AT)4 | 8 | 28466 | 28473 | LSC |  |
| 18 | C | 46 | 29220 | 29265 | LSC |  |
| 19 | (TA)4 | 8 | 31831 | 31838 | LSC |  |
| 20 | (AT)4 | 8 | 32775 | 32782 | LSC |  |
| 21 | (GA)4 | 8 | 36021 | 36028 | LSC | *trnS-UGA* |
| 22 | (TA)5 | 10 | 36251 | 36260 | LSC |  |
| 23 | C | 28 | 36984 | 37011 | LSC |  |
| 24 | (AT)4 | 8 | 45791 | 45798 | LSC |  |
| 25 | (TA)5 | 10 | 47117 | 47126 | LSC |  |
| 26 | (A)10 | 10 | 47339 | 47348 | LSC |  |
| 27 | (T)13 | 13 | 47713 | 47725 | LSC |  |
| 28 | (A)12 | 12 | 48037 | 48048 | LSC |  |
| 29 | (AT)5 | 28 | 48389 | 48416 | LSC |  |
| 30 | C | 16 | 49376 | 49391 | LSC |  |
| 31 | C | 56 | 50296 | 50351 | LSC |  |
| 32 | C | 89 | 52581 | 52669 | LSC |  |
| 33 | (T)10 | 10 | 55581 | 55590 | LSC | *atpB* |
| 34 | C | 89 | 56052 | 56140 | LSC |  |
| 35 | (T)17 | 17 | 60658 | 60674 | LSC |  |
| 36 | (AT)5 | 10 | 60839 | 60848 | LSC |  |
| 37 | (TC)5 | 10 | 62100 | 62109 | LSC | *cemA* |
| 38 | (AT)4 | 8 | 63006 | 63013 | LSC | *petA* |
| 39 | (T)13 | 13 | 64015 | 64027 | LSC |  |
| 40 | (G)13 | 13 | 64242 | 64254 | LSC |  |
| 41 | C | 27 | 64576 | 64602 | LSC |  |
| 42 | (TA)4 | 8 | 66500 | 66507 | LSC |  |
| 43 | (TA)4 | 8 | 67431 | 67438 | LSC |  |
| 44 | (T)10 | 10 | 68536 | 68545 | LSC |  |
| 45 | (A)12 | 12 | 69404 | 69415 | LSC |  |
| 46 | (AT)4 | 8 | 70824 | 70831 | LSC |  |
| 47 | C | 115 | 71305 | 71419 | LSC | *clpP* intron2 |
| 48 | (T)12 | 12 | 71566 | 71577 | LSC | *clpP* intron2 |
| 49 | C | 25 | 72576 | 72600 | LSC | *clpP* intron1 |
| 50 | (AT)4 | 8 | 73267 | 73274 | LSC |  |
| 51 | (AT)4 | 8 | 77587 | 77594 | LSC |  |
| 52 | (A)11 | 11 | 79030 | 79040 | LSC |  |
| 53 | (T)13 | 13 | 81731 | 81743 | LSC |  |
| 54 | (T)13 | 13 | 82241 | 82253 | LSC |  |
| 55 | (A)10 | 10 | 83077 | 83086 | LSC | *rpl16* intron |
| 56 | (T)10 | 10 | 83780 | 83789 | LSC |  |
| 57 | C | 43 | 84500 | 84542 | LSC |  |
| 58 | (TA)4 | 8 | 86026 | 86033 | IRB | *rpl2* intron |
| 59 | (GA4) | 20 | 87644 | 87663 | IRB | *ycf2* |
| 60 | (GA)4 | 8 | 88643 | 88650 | IRB | *ycf2* |
| 61 | (GA)4 | 8 | 90858 | 90865 | IRB | *ycf2* |
| 62 | (AG)4 | 8 | 96322 | 96329 | IRB | *ndhB* exon2 |
| 63 | (T)10 | 10 | 101012 | 101021 | IRB |  |
| 64 | (CT)4 | 8 | 107258 | 107265 | IRB | 23S rRNA |
| 65 | (AG)4 | 8 | 109102 | 109109 | IRB |  |
| 66 | (AT)4 | 8 | 110064 | 110071 | IRB | *ycf1* |
| 67 | (TAA)5 | 15 | 113575 | 113589 | SSC |  |
| 68 | (T)10 | 10 | 114399 | 114408 | SSC |  |
| 69 | (TA)4 | 8 | 116697 | 116704 | SSC |  |
| 70 | (AT)6 | 12 | 121008 | 121019 | SSC |  |
| 71 | C | 68 | 121836 | 121903 | SSC | *ndhA* intron |
| 72 | C | 74 | 122076 | 122149 | SSC | *ndhA* intron |
| 73 | (A)12 | 12 | 122756 | 122767 | SSC | *ndhA* intron |
| 74 | (T)10 | 10 | 125521 | 125530 | SSC | *ycf1* |
| 75 | (T)11 | 11 | 126599 | 126609 | SSC | *ycf1* |
| 76 | (T)10 | 10 | 127543 | 127552 | SSC | *ycf1* |
| 77 | (T)13 | 13 | 129184 | 129196 | SSC | *ycf1* |
| 78 | (AT)4 | 8 | 131175 | 131182 | IRA | *ycf1* |
| 79 | (CT)4 | 8 | 132137 | 132144 | IRA |  |
| 80 | (AG)4 | 8 | 133981 | 133988 | IRA | 23S rRNA |
| 81 | (A)10 | 10 | 140225 | 140234 | IRA |  |
| 82 | (CT)4 | 8 | 144917 | 144924 | IRA |  |
| 83 | (TC)4 | 8 | 150381 | 150388 | IRA | *ycf2* |
| 84 | (TC)4 | 8 | 152596 | 152603 | IRA | *ycf2* |
| 85 | (TC)4 | 20 | 153583 | 153602 | IRA | *ycf2* |
| 86 | (AT)4 | 8 | 155212 | 155219 | IRA |  |

A total of 25 SSRs (out of 111 copies) are identified as compound formation. “C” represent a compound repeats.
