# Supplementary material for: Comparison of the Whole-Plastome Sequence between the Bonin Islands Endemic Rubus boninensis and Its Close Relative, Rubus trifidus (Rosaceae), in the Southern Korean Peninsula
Source: Genes (Basel). 2019 Oct 2;10(10):774. doi: 10.3390/genes10100774 (PMC6826710; doi:10.3390/genes10100774)
Supplement: Supplementary file 1 [file genes-10-00774-s001.zip › Table S2.docx]

**Table S2.** Distribution, length, and location of repeat sequences in the *Rubus trifidus* plastome sequence.

| cpSSR ID | Repeat Motif | Length (bp) | Start | End | Region | Annotation |
| --- | --- | --- | --- | --- | --- | --- |
| 1 | (TA)4 | 8 | 1620 | 1627 | LSC |  |
| 2 | (T)11 | 11 | 2196 | 2206 | LSC | *matK/trnK* intron |
| 3 | (A)10 | 10 | 4294 | 4303 | LSC |  |
| 4 | (T)11 | 11 | 4462 | 4472 | LSC |  |
| 5 | (TA)4 | 8 | 4771 | 4778 | LSC |  |
| 6 | (A)10 | 10 | 6114 | 6123 | LSC | *rps16* intron |
| 7 | (AG)5 | 10 | 6810 | 6819 | LSC |  |
| 8 | C | 36 | 7177 | 7212 | LSC |  |
| 9 | (A)11 | 11 | 7947 | 7957 |  |  |
| 10 | C | 47 | 8707 | 8753 | LSC |  |
| 11 | (T)10 | 10 | 9940 | 9949 | LSC |  |
| 12 | (TTA)4 | 12 | 10404 | 10415 | LSC |  |
| 13 | (T)12 | 12 | 12274 | 12285 | LSC |  |
| 14 | (T)10 | 10 | 13985 | 13994 | LSC |  |
| 15 | (T)11 | 11 | 18085 | 18095 | LSC | *rpoC2* gene |
| 16 | (TA)5 | 10 | 19439 | 19448 | LSC | *rpoC2* gene |
| 17 | (T)10 | 10 | 22526 | 22535 | LSC | *rpcC1* intron |
| 18 | (T)10 | 10 | 25787 | 25796 | LSC | *rpoB* gene |
| 19 | (AT)4 | 8 | 26186 | 26193 | LSC | *rpoB* gene |
| 20 | (T)11 | 11 | 28314 | 28324 | LSC |  |
| 21 | (AT)4 | 8 | 28478 | 28485 | LSC |  |
| 22 | C | 46 | 29231 | 29276 | LSC |  |
| 23 | (TC)4 | 8 | 30471 | 30478 | LSC |  |
| 24 | (TA)4 | 8 | 31842 | 31849 | LSC |  |
| 25 | (GA)4 | 8 | 35997 | 36004 | LSC |  |
| 26 | (TA)5 | 10 | 36227 | 36236 | LSC |  |
| 27 | C | 28 | 36960 | 36987 | LSC |  |
| 28 | (A)11 | 11 | 44906 | 44916 | LSC | *ycf3* intron 1 |
| 29 | (AT)4 | 8 | 45760 | 45767 | LSC |  |
| 30 | (TCCTAA)3 | 18 | 46147 | 46164 | LSC |  |
| 31 | (TA)5 | 10 | 47092 | 47101 | LSC |  |
| 32 | (T)13 | 13 | 47693 | 47705 | LSC |  |
| 33 | C | 28 | 48381 | 48408 | LSC |  |
| 34 | C | 16 | 49368 | 49383 | LSC |  |
| 35 | C | 55 | 50288 | 50342 | LSC |  |
| 36 | C | 90 | 52572 | 52661 | LSC |  |
| 37 | (T)10 | 10 | 55573 | 55582 | LSC | *atpB* gene |
| 38 | C | 90 | 56044 | 56133 | LSC |  |
| 39 | (T)12 | 12 | 60676 | 60687 | LSC |  |
| 40 | (AT)5 | 10 | 60852 | 60861 | LSC |  |
| 41 | (TC)5 | 10 | 62118 | 62127 | LSC | *cemA* gene |
| 42 | (AT)4 | 8 | 63024 | 63031 | LSC | *petA* gene |
| 43 | (T)13 | 13 | 64033 | 64045 | LSC |  |
| 44 | (G)11 | 11 | 64260 | 64270 | LSC |  |
| 45 | C | 17 | 64602 | 64618 | LSC |  |
| 46 | (TA)4 | 8 | 66515 | 66522 | LSC |  |
| 47 | (TA)4 | 8 | 67446 | 67453 | LSC |  |
| 48 | (T)12 | 12 | 67871 | 67882 | LSC |  |
| 49 | (T)10 | 10 | 68551 | 68560 | LSC |  |
| 50 | (A)12 | 12 | 69419 | 69430 | LSC |  |
| 51 | (AT)4 | 8 | 70839 | 70846 | LSC |  |
| 52 | C | 117 | 71317 | 71433 | LSC | *clpP* intron2 |
| 53 | (T)12 | 12 | 71580 | 71591 | LSC | *clpP* intron2 |
| 54 | (T)11 | 11 | 72598 | 72608 | LSC | *clpP* intron 1 |
| 55 | (AT)4 | 8 | 73293 | 73300 | LSC |  |
| 56 | (AAT)4 | 12 | 76513 | 76524 | LSC | *petB* intron |
| 57 | (AT)4 | 8 | 77613 | 77620 | LSC |  |
| 58 | (A)13 | 13 | 79056 | 79068 | LSC |  |
| 59 | (T)14 | 14 | 81759 | 81772 | LSC |  |
| 60 | (T)12 | 12 | 82270 | 82281 | LSC |  |
| 61 | (A)10 | 10 | 83111 | 83120 | LSC | *rps16* intron |
| 62 | (T)11 | 11 | 83815 | 83825 | LSC |  |
| 63 | C | 43 | 84536 | 84578 | LSC |  |
| 64 | (TA)4 | 8 | 86054 | 86061 | IRB | *rpl2* intron |
| 65 | C | 20 | 87672 | 87691 | IRB |  |
| 66 | (GA)4 | 8 | 88671 | 88678 | IRB | *ycf2* gene |
| 67 | (GA)4 | 8 | 90886 | 90893 | IRB | *ycf2* gene |
| 68 | (AG)4 | 8 | 96350 | 96357 | IRB | *ndhB* exon2 |
| 69 | (CT)4 | 8 | 107292 | 107299 | IRB | 23s rRNA gene |
| 70 | (AG)4 | 8 | 109136 | 109143 | IRB |  |
| 71 | (AT)4 | 8 | 110098 | 110105 | IRB |  |
| 72 | C | 88 | 113537 | 113624 | SSC |  |
| 73 | C | 31 | 114857 | 114887 | SSC |  |
| 74 | (TA)4 | 8 | 116718 | 116725 | SSC |  |
| 75 | (AT)7 | 14 | 121025 | 121038 | SSC |  |
| 76 | (AT)4 | 8 | 121330 | 121337 | SSC | *ndhA* exon2 |
| 77 | C | 62 | 121855 | 121916 | SSC | *ndhA* intron |
| 78 | (TA)4 | 76 | 122095 | 122170 | SSC | *ndhA* intron |
| 79 | (A)13 | 13 | 122777 | 122789 | SSC | *ndhA* intron |
| 80 | (T)10 | 10 | 125534 | 125543 | SSC | *ycf1* gene |
| 81 | (T)11 | 11 | 126612 | 126622 | SSC | *ycf1* gene |
| 82 | (T)10 | 10 | 127556 | 127565 | SSC | *ycf1* gene |
| 83 | (T)10 | 10 | 129197 | 129206 | SSC | *ycf1* gene |
| 84 | (AT)4 | 8 | 131185 | 131192 | IRA |  |
| 85 | (CT)4 | 8 | 132147 | 132154 | IRA |  |
| 86 | (AG)4 | 8 | 133991 | 133998 | IRA | 23S rRNA gene |
| 87 | (CT)4 | 8 | 144933 | 144940 | IRA | *ndhB* exon2 |
| 88 | (TC)4 | 8 | 150397 | 150404 | IRA | *ycf2* gene |
| 89 | (TC)4 | 8 | 152612 | 152619 | IRA | *ycf2* gene |
| 90 | C | 20 | 153599 | 153618 | IRA | *ycf2* gene |
| 91 | (AT)4 | 8 | 155228 | 155235 | IRA | *rpl2* exon2 |

A total of 21 SSRs (out of 112 copies) are identified as compound formation. “C” represent a compound repeats.
