# Supplementary material for: Comparison of the Whole-Plastome Sequence between the Bonin Islands Endemic Rubus boninensis and Its Close Relative, Rubus trifidus (Rosaceae), in the Southern Korean Peninsula
Source: Genes (Basel). 2019 Oct 2;10(10):774. doi: 10.3390/genes10100774 (PMC6826710; doi:10.3390/genes10100774)
Supplement: Supplementary file 1 [file genes-10-00774-s001.zip › Table S3.docx]

**Table S3.** Distribution, length, and location of repeat sequences in the *Rubus crataegifolius* plastome sequence.

| cpSSR ID | Repeat Motif | Length (bp) | Start | End | Region | Annotation |
| --- | --- | --- | --- | --- | --- | --- |
| 1 | (A)11 | 11 | 223 | 233 | LSC |  |
| 2 | (TA)4 | 8 | 1672 | 1679 | LSC |  |
| 3 | (T)11 | 11 | 2246 | 2256 | LSC | *matK/trnK* intron |
| 4 | (A)10 | 10 | 4344 | 4353 | LSC |  |
| 5 | (T)10 | 10 | 4518 | 4527 | LSC |  |
| 6 | (TA)4 | 8 | 4843 | 4850 | LSC |  |
| 7 | (C)10 | 10 | 6216 | 6225 | LSC | *rps16* intron |
| 8 | (T)10 | 10 | 6543 | 6552 | LSC |  |
| 9 | C | 108 | 6869 | 6976 | LSC |  |
| 10 | C | 70 | 7232 | 7301 | LSC |  |
| 11 | (A)12 | 12 | 8785 | 8796 | LSC |  |
| 12 | (TTA)4 | 12 | 10454 | 10465 | LSC |  |
| 13 | (T)11 | 11 | 12307 | 12317 | LSC |  |
| 14 | (T)11 | 11 | 14024 | 14034 | LSC |  |
| 15 | (T)11 | 11 | 18105 | 18115 | LSC | *rpoC2* gene |
| 16 | (TA)5 | 10 | 19468 | 19477 | LSC | *rpoC2* gene |
| 17 | (T)10 | 10 | 25815 | 25824 | LSC | *rpoB* gene |
| 18 | (AT)4 | 8 | 26214 | 26221 | LSC | *rpoB* gene |
| 19 | (T)10 | 10 | 28346 | 28355 | LSC |  |
| 20 | (AT)4 | 8 | 28526 | 28533 | LSC |  |
| 21 | C | 64 | 29263 | 29326 | LSC |  |
| 22 | (TA)4 | 8 | 31855 | 31862 | LSC |  |
| 23 | (T)11 | 11 | 32371 | 32381 | LSC |  |
| 24 | (TA)4 | 8 | 32611 | 32618 | LSC |  |
| 25 | C | 90 | 32770 | 32859 | LSC |  |
| 26 | (GA)4 | 8 | 35990 | 35997 | LSC | *trnS-tRNA* |
| 27 | (TA)5 | 10 | 36220 | 36229 | LSC |  |
| 28 | (TA)4 | 8 | 36976 | 36983 | LSC |  |
| 29 | (A)11 | 11 | 44905 | 44915 | LSC | *ycf3* intron 1 |
| 30 | (AT)4 | 8 | 45731 | 45738 | LSC |  |
| 31 | (A)15 | 15 | 47310 | 47324 | LSC |  |
| 32 | (T)10 | 10 | 47697 | 47706 | LSC |  |
| 33 | C | 130 | 47881 | 48010 | LSC |  |
| 34 | C | 28 | 48346 | 48373 | LSC |  |
| 35 | C | 16 | 49334 | 49349 | LSC |  |
| 36 | C | 56 | 50245 | 50300 | LSC |  |
| 37 | (T)11 | 11 | 51694 | 51704 | LSC |  |
| 38 | C | 36 | 52541 | 52576 | LSC |  |
| 39 | (T)10 | 10 | 55547 | 55556 | LSC | *atpB* gene |
| 40 | (AT)4 | 8 | 56099 | 56106 | LSC |  |
| 41 | (T)12 | 12 | 60578 | 60589 | LSC |  |
| 42 | (AT)5 | 10 | 60754 | 60763 | LSC |  |
| 43 | (T)10 | 10 | 61669 | 61678 | LSC |  |
| 44 | (TC)5 | 10 | 62026 | 62035 | LSC | *cemA* gene |
| 45 | (AT)4 | 8 | 62932 | 62939 | LSC | *petA* gene |
| 46 | (T)13 | 13 | 63941 | 63953 | LSC |  |
| 47 | (G)13 | 13 | 64170 | 64182 | LSC |  |
| 48 | C | 17 | 64520 | 64536 | LSC |  |
| 49 | (T)10 | 10 | 64675 | 64684 | LSC |  |
| 50 | (TA)4 | 8 | 66400 | 66407 | LSC |  |
| 51 | (TA)4 | 8 | 67331 | 67338 | LSC |  |
| 52 | (T)10 | 10 | 68449 | 68458 | LSC |  |
| 53 | C | 28 | 69321 | 69348 | LSC |  |
| 54 | (T)11 | 11 | 70498 | 70508 | LSC |  |
| 55 | (AT)4 | 8 | 70750 | 70757 | LSC |  |
| 56 | (T)11 | 11 | 71488 | 71498 | LSC | *clpP* intron2 |
| 57 | (C)10(T)11 | 21 | 72498 | 72518 | LSC | *clpP* intron1 |
| 58 | (AT)4 | 8 | 73193 | 73200 | LSC |  |
| 59 | (AAT)4 | 12 | 76413 | 76424 | LSC | *petB* intron1 |
| 60 | (AT)4 | 8 | 77513 | 77520 | LSC |  |
| 61 | (A)10 | 10 | 78967 | 78976 | LSC |  |
| 62 | (T)13 | 13 | 81663 | 81675 | LSC |  |
| 63 | (T)11 | 11 | 82171 | 82181 | LSC |  |
| 64 | C | 121 | 82971 | 83091 | LSC | *rpl16* intron1 |
| 65 | C | 62 | 83460 | 83521 | LSC | *rpl16* intron1 |
| 66 | C | 43 | 84452 | 84494 | LSC |  |
| 67 | C | 25 | 85048 | 85072 | LSC |  |
| 68 | (TA)4 | 8 | 85990 | 85997 | IRB | *rpl2* intron |
| 69 | C | 20 | 87608 | 87627 | IRB | *ycf2* gene |
| 70 | (GA)4 | 8 | 88607 | 88614 | IRB | *ycf2* gene |
| 71 | (GA)4 | 8 | 90822 | 90829 | IRB | *ycf2* gene |
| 72 | (AG)4 | 8 | 96291 | 96298 | IRB | *ndhB* gene |
| 73 | (T)10 | 10 | 100962 | 100971 | IRB |  |
| 74 | (CT)4 | 8 | 107210 | 107217 | IRB | 23S rRNA gene |
| 75 | (AG)4 | 8 | 109054 | 109061 | IRB |  |
| 76 | (AT)4 | 8 | 110016 | 110023 | IRB |  |
| 77 | (A)11 | 11 | 114886 | 114896 | SSC |  |
| 78 | (TA)4 | 8 | 116630 | 116637 | SSC |  |
| 79 | (AT)6 | 12 | 120947 | 120958 | SSC |  |
| 80 | (A)10 | 10 | 121823 | 121832 | SSC | *ndhA* intron |
| 81 | C | 77 | 122009 | 122085 | SSC | *ndhA* intron |
| 82 | (A)12 | 12 | 122696 | 122707 | SSC | *ndhA* intron |
| 83 | (T)10 | 10 | 124927 | 124936 | SSC |  |
| 84 | (T)10 | 10 | 125461 | 125470 | SSC | *ycf1* gene |
| 85 | (T)11 | 11 | 126524 | 126534 | SSC | *ycf1* gene |
| 86 | (T)12 | 12 | 129103 | 129114 | SSC | *ycf1* gene |
| 87 | (AT)4 | 8 | 131094 | 131101 | IRA |  |
| 88 | (CT)4 | 8 | 132056 | 132063 | IRA |  |
| 89 | (AG)4 | 8 | 133900 | 133907 | IRA | 23S rRNA gene |
| 90 | (A)10 | 10 | 140146 | 140155 | IRA |  |
| 91 | (CT)4 | 8 | 144819 | 144826 | IRA | *ndhB* gene |
| 92 | (TC)4 | 8 | 150288 | 150295 | IRA | *ycf2* gene |
| 93 | (TC)4 | 8 | 152503 | 152510 | IRA | *ycf2* gene |
| 94 | C | 20 | 153490 | 153509 | IRA | *ycf2* gene |
| 95 | (AT)4 | 8 | 155119 | 155126 | IRA | *rpl2* intron |

A total of 27 SSRs (out of 122 copies) are identified as compound formation. “C” represent a compound repeats.
